# Supplementary material for: A set of multi-entry identification keys to African frugivorous flies (Diptera, Tephritidae)
Source: Zookeys. 2014 Jul 24;(428):97–108. doi: 10.3897/zookeys.428.7366 (PMC4143993; doi:10.3897/zookeys.428.7366)
Supplement: Supplementary material 4 — Key to Capparimyia [file zookeys-428-097-s004.zip › SF4_ZooKeys_key to Capparimyia/lucid_player/help/help/whgdata/whlstf2.htm]

Search Words List


**<<**

**error**   
**errors**   
**essentially**   
etc **1** **2** **3** **4** **5**   
**even**   
example **1** **2** **3** **4** **5** **6** **7** **8** **9** **10** **11**   
**executed**   
**existing**   
**exit**   
**expanding**   
**experienced**   
expert **1** **2** **3**   
**explanatory**   
**extended**   
extra **1** **2**   
  
  
**f**   
fact **1** **2** **3**   
**families**   
family **1** **2**   
faq **1** **2**   
**faq\_lucid3**   
feature **1** **2** **3** **4** **5** **6** **7** **8** **9** **10** **11** **12** **13** **14** **15** **16** **17** **18** **19** **20**   
features **1** **2** **3** **4** **5** **6** **7** **8** **9** **10** **11** **12** **13** **14** **15** **16** **17** **18** **19** **20** **21**   
**feel**   
file **1** **2**   
files **1** **2** **3** **4**   
**filter**   
filtered **1** **2**   
**finally**   
find **1** **2** **3** **4** **5** **6** **7**   
finding **1** **2** **3** **4** **5**   
first **1** **2** **3**   
**fish**   
**flavour**   
**flexibility**   
**flexible**   
flies **1** **2**   
flower **1** **2** **3**   
flowers **1** **2**   
**fly**   
**focus**   
folder **1** **2**   
**folders**   
**follow**   
following **1** **2**   
**follows**   
font **1** **2**   
**fonts**   
**forewing**   
**forms**   
**four**   
free **1** **2** **3**   
**frogs**   
fruit **1** **2** **3** **4**   
**fruiting**   
fruits **1** **2**   
function **1** **2** **3** **4** **5** **6** **7** **8** **9**   
functions **1** **2** **3** **4** **5**   
**further**   
  
  
**g**   
gallery **1** **2** **3** **4**   
**genera**   
**general**   
get **1** **2**   
**getting**   
given **1** **2** **3**   
good **1** **2**   
**grasses**   
greater **1** **2** **3** **4** **5**   
group **1** **2**   
**grouping**   
groups **1** **2**   
**guides**   
  
  
**half**   
**halved**   
**handled**   
**handles**   
**handy**   
**hat**   
having **1** **2**   
**hd**   
**head**   
**headings**   
help **1** **2** **3** **4** **5** **6** **7** **8** **9**   
hence **1** **2** **3**   
**hidden**   
hierarchical **1** **2** **3**   
**hierarchy**   
**hints**   
hold **1** **2** **3**   
**home**   
**honeybee**   
how **1** **2** **3** **4** **5**   
**htm**   
html **1** **2**   
**http**   
  
  
**icon**   
**icons**   
identification **1** **2** **3** **4** **5** **6** **7** **8** **9** **10** **11**   
**identifications**   
identified **1** **2**   
identify **1** **2** **3** **4** **5**   
**identifying**   
if **1** **2** **3** **4** **5** **6** **7** **8** **9** **10** **11** **12** **13** **14** **15** **16** **17** **18** **19**   
**ignored**   
**ignoring**   
**illustrated**   
image **1** **2** **3**   
images **1** **2** **3** **4** **5**   
**immediate**   
**immediately**   
implementation **1** **2**   
**important**   
**included**   
**includes**   
**including**   
**incorrectly**   
**increase**   
**index**   
indicated **1** **2**   
information **1** **2** **3** **4** **5** **6** **7** **8** **9** **10** **11**   
**insects**   
install **1** **2** **3**   
**installation**   
installed **1** **2** **3** **4**   
**installing**   
**instance**   
instead **1** **2**   
**integer**   
**intended**   
interactive **1** **2** **3**   
**interchangeably**   
**interest**   
**interested**   
**interface**   
**internationalisation**   
**introduction**   
**invoked**

**>>**
